# *Thermoascus aurantiacus* is a promising source of enzymes for biomass deconstruction under thermophilic conditions

**DOI:** 10.1186/1754-6834-5-54

**Published:** 2012-07-28

**Authors:** Shara D McClendon, Tanveer Batth, Christopher J Petzold, Paul D Adams, Blake A Simmons, Steven W Singer

**Affiliations:** 1 Joint BioEnergy Institute, Emeryville, CA, USA; 2 Physical Biosciences Division, Lawrence Berkeley National Laboratory, Berkeley, CA, USA; 3 Biomass Science and Conversion Technology Department, Sandia National Laboratories, Livermore, CA, USA; 4 Earth Sciences Division, Lawrence Berkeley National Laboratory, Berkeley, CA, USA; 5 Deconstruction Division, Joint BioEnergy Institute, 5885 Hollis Street, Emeryville, CA, 94608, USA

**Keywords:** *Thermoascus aurantiacus*, *Thielavia terrestris*, GH 61, Polysaccharide monooxygenases, Fungal secretome, Ammonia fiber expansion, Ionic liquid, 1-ethyl-3-methylimidazolium acetate, Switchgrass (*Panicum virgatum*)

## Abstract

**Background:**

Thermophilic fungi have attracted increased interest for their ability to secrete enzymes that deconstruct biomass at high temperatures. However, development of thermophilic fungi as enzyme producers for biomass deconstruction has not been thoroughly investigated. Comparing the enzymatic activities of thermophilic fungal strains that grow on targeted biomass feedstocks has the potential to identify promising candidates for strain development. *Thielavia terrestris* and *Thermoascus aurantiacus* were chosen for characterization based on literature precedents.

**Results:**

*Thermoascus aurantiacus* and *Thielavia terrestris* were cultivated on various biomass substrates and culture supernatants assayed for glycoside hydrolase activities. Supernatants from both cultures possessed comparable glycoside hydrolase activities when incubated with **artificial** biomass substrates. In contrast, saccharifications of ionic liquid pretreated switchgrass (*Panicum virgatum*) revealed that *T. aurantiacus* enzymes released more glucose than *T. terrestris* enzymes over a range of protein mass loadings and temperatures. Temperature-dependent saccharifications demonstrated that the *T. aurantiacus* proteins retained higher levels of activity compared to a commercial enzyme mixture sold by Novozymes, Cellic CTec2, at elevated temperatures. Enzymes secreted by *T. aurantiacus* released glucose at similar protein loadings to CTec2 on dilute acid, ammonia fiber expansion, or ionic liquid pretreated switchgrass. Proteomic analysis of the *T. aurantiacus* culture supernatant revealed dominant glycoside hydrolases from families 5, 7, 10, and 61, proteins that are key enzymes in commercial cocktails.

**Conclusions:**

*T. aurantiacus* produces a complement of secreted proteins capable of higher levels of saccharification of pretreated switchgrass than *T. terrestris* enzymes. The *T. aurantiacus* enzymatic cocktail performs at the same level as commercially available enzymatic cocktail for biomass deconstruction, without strain development or genetic modifications. Therefore, *T. aurantiacus* provides an excellent **platform** to develop a thermophilic fungal system for enzyme production for the conversion of biomass to biofuels.

## Background

The secretomes of thermophilic fungi have been recently identified as promising sources of enzymes for improving the biochemical conversion of lignocellulosic biomass to biofuels [1]. Readily culturable thermophilic fungi are saprophytic and found in a range of environments, such as compost, where decomposition of organic matter occurs at elevated temperatures [2]. Glycoside hydrolase (GH) enzymes from thermophilic fungi function from 60–80°C [1,3-5], whereas commercial fungal cocktails produced by mesophilic fungi perform optimally at 50°C. Performing saccharifications of pretreated biomass at elevated temperatures contributes to increased conversion rates [6] resulting in shorter incubation times and lower enzyme loadings, as well as reducing the risk of downstream contamination by competing microorganisms for fermentable sugars. Therefore, thermophilic fungi may provide new enzymatic cocktails that are optimized for the industrial biochemical conversion of biomass to sugars for fermentation to biofuels.

Prior to fermentation, lignocellulosic biomass (e.g., corn stover, switchgrass) is typically pretreated chemically to reduce its recalcitrant properties. Pretreatment increases the efficiency of subsequent enzymatic hydrolysis of plant polysaccharides. Certain ionic liquids (ILs) have shown excellent promise in generating cellulose from biomass that readily hydrolyzed by enzymatic cocktails [7]. The majority of ILs that are effective biomass solvents are also toxic to microbes, however fungi have been shown to grow in the presence of high **concentrations** of some ILs [8]. Recently, a strain of the thermotolerant fungus *Aspergillus fumigatus* was grown on switchgrass in the presence of 5% 1-butyl-3-methylimidazolium chloride, abbreviated as [C_4_mim]Cl, secreting high **amounts** of cellulase and hemicellulase enzymes. These GHs were shown to retain residual activity in up to 20% [C_4_mim]Cl [9]. In addition, increased IL tolerance has been demonstrated for thermophilic glycoside hydrolases from bacteria [10,11] and archaea [12], suggesting a **correlation** between thermotolerance and IL tolerance. Therefore, thermophilic fungal enzymes may express enzymes that are tolerant to residual IL present in pretreated biomass, enabling a more cost-effective IL conversion process with minimal washing required after pretreatment.

In addition, culture supernatants of thermophilic fungi have been instrumental in identifying new accessory proteins that improve cellulose hydrolysis. Increased glucose release from pretreated barley straw was observed with mixtures of Cellulclast and Novozymes 188 cocktails supplemented with thermophilic fungal crude extracts, indicating that thermophilic fungi produce enhancers for cellulose hydrolysis [13]. Biochemical and proteomic studies identified these enhancement factors as proteins that are members of GH family 61. Addition of purified GH61 proteins from thermophilic fungi *Thielavia terrestris* or *Thermoascus aurantiacus* to a commercial *Trichoderma reesei* enzyme cocktail gave improved cellulose hydrolysis, resulting in 2-fold lower protein loading requirements [14]. Detailed characterization of GH61 from *Thermoascus aurantiacus* demonstrated that these proteins are Cu-containing monoxygenases that oxidize cellulose chains, facilitating glycoside hydrolase depolymerization [15]. These discoveries **demonstrate** the potential for studying thermophilic fungi as sources of proteins to improve hydrolysis of lignocellulosic biomass.

Our aim is to gain an understanding of the mechanisms that thermophilic fungi use to deconstruct biomass and develop industrially relevant enzyme cocktails for saccharification of pretreated biomass. Previous studies of thermophilic fungi have indicated that *Thielavia terrestris* and *Thermoascus aurantiacus* are promising species for the production of a thermophilic enzymatic cocktail for a lignocellulose deconstruction [13]. In this work, enzymatic hydrolytic activities of supernatants from *T. aurantiacus* and *T. terrestris* grown on untreated and chemically pretreated biomass were compared. This comparison, performed on **artificial** biomass substrates and pretreated biomass, established *T. aurantiacus* as a promising system to develop thermophilic enzymatic cocktails.

## Results

### Growth of thermophilic fungi on biomass substrates

*Thielavia terrestis* NRRL 8126 and *Thermoascus aurantiacus***ATCC** 26904 were cultured on ethanol-extracted switchgrass (SG), ammonia fiber expansion treated switchgrass (AFEX-SG), 1-ethyl-3-methylimidazolium acetate ([C_2_mim][OAc]) treated switchgrass (IL-SG), and microcrystalline cellulose (MCC, **Sigma #435244**) for 3 days at 50°C. Culture supernatants were separated from fungal mycelia and remaining solids by filtration. The protein concentrations of the supernatants were estimated using both Bradford and BCA methods (Table 1). Since the BCA method is sensitive to the reducing sugars present in the supernatant, the proteins were precipitated using acetone to remove components that may interfere with the protein assay [16]. Values using either assay were comparable, except those generated with AFEX-SG; the higher levels of protein observed by SDS-PAGE in the supernatants from the AFEX-SG grown cultures were more consistent with the estimates obtained for the Bradford assay (see below). 

**Table 1:** **Estimated protein concentrations of culture supernatants using two protein assay methods**^**1**^

**Strain**	**Carbon source**	**Bradford method (mg/mL)**	**BCA method (mg/mL)**
*Thermoascus aurantiacus*	SG	1.0 ± 0.2	1.1 ± 0.1
	AFEX SG	5.7 ± 0.2	2.1 ± 0.1
	IL SG	2.1 ± 0.1	2.0 ± 0.1
	MCC	0.9 ± 0.3	-
*Thielavia terrestris*	SG	0.8 ± 0.2	1.1 ± 0.1
	AFEX SG	3.1 ± 0.1	1.3 ± 0.1
	IL SG	1.5 ± 0.2	1.0 ± 0.1
	MCC	1.4 ± 0.2	-

^1^ Three replicates were performed and the error bars represent standard deviation from the mean.

### Supernatant activities on artificial substrates

Cellulase (endoglucanase, cellobiohydrolyase, beta-glucosidase) and hemicelluase (xylanase, beta-xylosidase, arabinofuranosidase) activities were measured for these supernatants using **artificial** substrates (Table 2). Comparison of the cellulase profiles indicated that higher levels of endoglucanase was present in the *T. terrestris* supernatant but the *T. aurantiacus* supernatant possesses higher levels of cellobiohydrolase and β-glucosidase activity. For hemicellulosic substates, xylanase activity was higher for *T. aurantiacus* but lower for β-xylosidase activity. In general, the highest activities for the **artificial** biomass substrates were observed on supernatants generated from growth with SG and MCC.

**Table 2:** **Secreted cellulase and hemicellulase activities (U/mg or mU/mg) from thermophilic fungi grown on complex biomass substrates**^**1,2,3**^

**Strain/Carbon source**	**CMC (U/mg)**	**pNPC (mU/mg)**	**pNPG (mU/mg)**	**Xylan (U/mg)**	**pNPX (mU/mg)**
*T. aurantiacus*					
Switchgrass	0.17 ± 0.03	34.4 ± 1.31	177.3 ± 2.71	0.67 ± 0.01	8.8 ± 0.81
AFEX Switchgrass	0.04 ± 0.01	25.7 ± 0.40	45.6 ± 1.71	0.10 ± 0.02	12.4 ± 1.63
IL Switchgrass	0.02 ± 0.01	9.8 ± 0.26	54.8 ± 0.43	0.35 ± 0.01	10.3 ± 1.09
Microcrystalline Cellulose	1.94 ± 0.22	12.5 ± 1.60	93.8 ± 12.86	1.90 ± 0.62	6.92 ± 0.49
*T. terrestris*					
Switchgrass	0.79 ± 0.02	11.3 ± 0.62	26.1 ± 1.14	2.44 ± 0.38	5.7 ± 0.47
AFEX Switchgrass	0.24 ± 0.01	3.7 ± 0.07	5.7 ± 1.21	0.66 ± 0.05	0.8 ± 0.37
IL Switchgrass	0.23 ± 0.02	7.8 ± 0.25	5.7 ± 0.23	1.02 ± 0.05	3.8 ± 0.41
Microcrystalline Cellulose	1.24 ± 0.23	16.2 ± 4.38	6.9 ± 1.76	3.27 ± 1.12	4.17 ± 1.27

^1^Specific activities based on protein concentrations obtained using the Bradford method.

^2^ Three replicates were performed and the error bars represent standard deviation from the mean.

^3^ Activities represented by each substrate is described in the Methods section.

**Figure 1:**
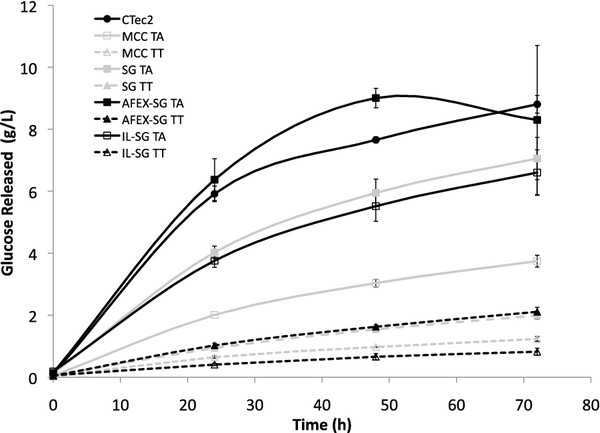
**Release of glucose from 2% [C**_**2**_**mim][OAc]-pretreated switchgrass. In the reactions, *T. aurantiacus *and *T. terrestris *culture supernatants were normalized to 1U CMCase activity.** Glucan conversion (based on glucose only): 7 – 68%. Three replicates were performed and the error bars represent standard deviation from the mean.

The eight culture supernatants were screened for glucose release on lignocellulosic biomass **substrate**; these tests were performed to determine the most active samples from each fungal strain. The enzymatic activities were compared to a commercially available enzyme cocktail, Cellic CTec2 (Novozymes), in the saccharification of **[C**_**2**_**mim][OAc]-treated switchgrass**. Time course data on **[C**_**2**_**mim][OAc]-treated switchgrass** demonstrated that *T. aurantiacus* enzymes performed comparably to CTec2 at 50°C, while *T. terrestris* enzymes released between **considerably** less glucose compared to the *T. aurantiacus* supernatant (**Figure**1). For each strain, supernatants **prepared** from cultures on AFEX-SG were the most active of all the samples in cellulose hydrolysis, followed by supernatants from untreated switchgrass (SG), [C_2_mim][OAc]-treated switchgrass (IL-SG), and microcrystalline cellulose (MCC), respectively.

### Saccharification with culture supernatants from thermophilic fungi

**Figure 2:**
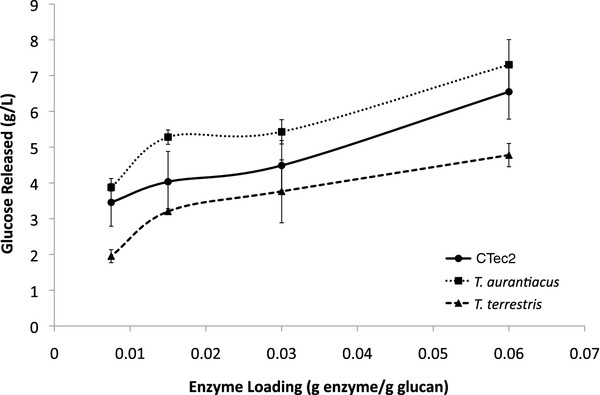
**Glucose released from [C**_**2**_**mim][OAc]-treated switchgrass at different enzyme loadings for culture supernatants from AFEX-SG grown *T. aurantiacus *and *T. terrestris *compared to equivalent enzyme loading from Cellic CTec2.** The reactions were performed at 60°C with 2% (w/v) [C_2_mim][OAc]-pretreated switchgrass. Three replicates were performed and the error bars represent standard deviation from the mean.

**Figure 3:**
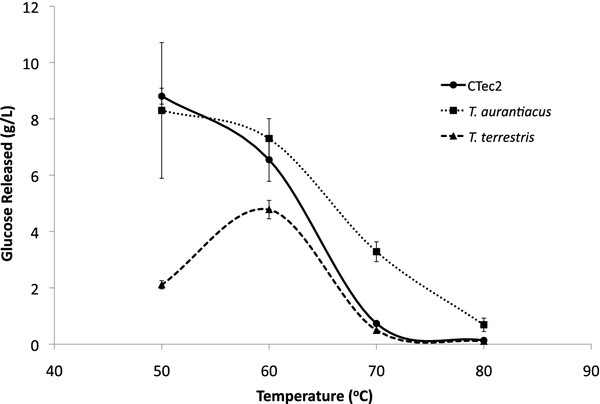
**Temperature profile of the saccharification of [C**_**2**_**mim][OAc]-pretreated switchgrass with *T. aurantiacus*/*T. terrestris *culture supernatants and Cellic CTec2.** Enzyme loading was set at 0.06 g enzyme/g glucan and biomass loading was 2% [C_2_mim][OAc]-pretreated switchgrass. Three replicates were performed and the error bars represent standard deviation from the mean.

The saccharification **experiment** described above established AFEX-grown cultures of *T. aurantiacus* and *T. terrestris* as promising sources of enzymes from thermophilic fungi. [C_2_mim][OAc] treated switchgrass (IL-SG) was used as a substrate, which has been shown to be an excellent substrate for commercial enzymatic cocktails [7]. Comparable amounts of glucose were released from IL-SG from *T. aurantiacus* enzymes and CTec2 at a range of protein loadings (Figure 2). **Activities** from *T. terrestris* were consistently lower at all protein loading levels. These trends were also observed when saccharifications were performed at different temperatures (Figure 3). The CTec2 cocktail and the AFEX-SG grown *T. aurantiacus* supernatant had the highest relative activity at 50°C; however the *T. aurantiacus* supernatant released almost 3-fold more glucose from IL-SG at 70°C than CTec2 and the *T. terrestris* supernatant. In contrast, the AFEX-SG *T. terrestris* supernatant had an activity maximum at 60°C, but the *T. aurantiacus* enzymes were 2-fold more active at the same temperature. 

**Figure 4:**
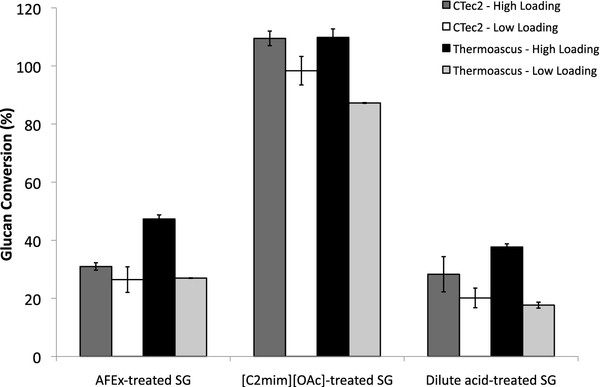
**Conversion of glucan to glucose and cellobiose using culture supernatants from AFEX-SG grown *T. aurantiacus *and Cellic CTec2 on various pretreated biomass substrates.** Reaction conditions were: (2% w/v) biomass, 60°C, pH 5 for 72 hours with mixing. Switchgrass substrates were pretreated in three different ways: IL – ionic liquid, [C_2_mim][OAc]; DA – dilute acid, H_2_SO_4_; AFEX-, NH_4_. The saccharification reactions were performed at two different enzyme loadings, corresponding to recommended high and low enzyme loadings for Cellic Ctec2 (http://www.bioenergy.novozymes.com/). Equal amounts of protein from both *T. aurantiacus* supernatants and Cellic CTec2 were added to each reaction (g enzyme/g glucan): AFEX-high/low ∼ 0.065/0.016; IL-high/low ∼ 0.046/0.011; DA-high/low ∼ 0.043/0.010. Three replicates were performed and the error bars represent standard deviation from the mean.

Saccharifications of this supernatant were the tested against dilute acid and AFEX-SG, which are more recalcitrant substrates compared to IL-SG [7,17]. Comparison of saccharification of dilute acid and AFEX-SG switchgrass by the *T. aurantiacus* and CTec2 indicated that at high enzyme loadings, more glucose was released by the *T. aurantiacus* supernatant compared to the CTec2 cocktail for all three substrates (Figure 4). High and low enzyme loadings were applied as suggested by CTec2 recommendations, with 1 – 6% (w/w) enzyme to cellulose representing the range of low to high enzyme concentrations. 

### Proteomic analysis of the *T. aurantiacus* secretome

**Figure 5:**
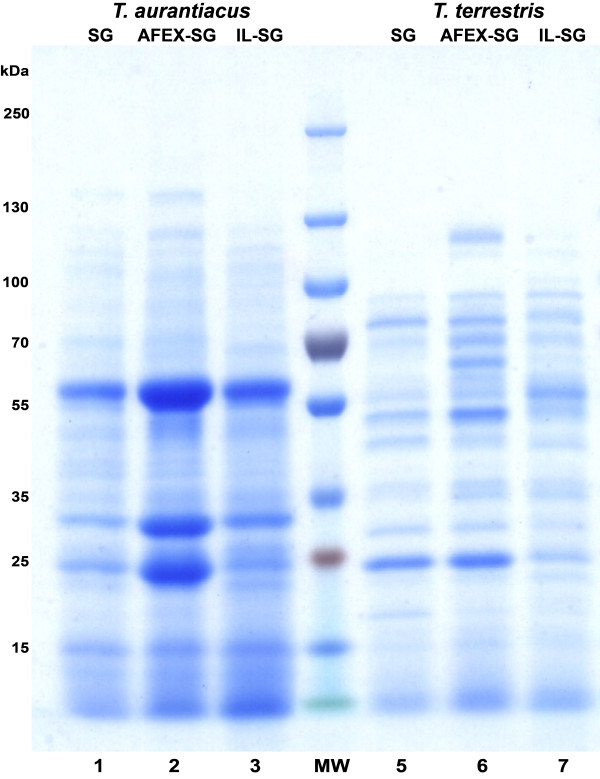
**SDS-PAGE analysis of supernatants from*T. aurantiacus *and *T. terrestris *on 8-16% Tricine polyacrylamide gels.** Lane 1, SG-grown *T. aurantiacus*; lane 2, AFEX-SG grown *T. aurantiacus*; lane 3, IL-SG grown *T. aurantiacus*; lane 4, MW markers; lane 5, SG-grown *T. terrestris*; lane 6, AFEX-SG grown *T. terrestris*; lane 7, IL-SG *T. terrestris.*

Proteomic analysis of the *T. aurantaicus* supernatants was performed to determine abundant proteins that may be responsible for the observed saccharification activity. Analysis of the *T. aurantiacus* supernatants showed distinct protein band patterns for each cultivation condition on untreated and pretreated switchgrass (Figure 5). The AFEX-SG supernatant had three prominent protein bands (∼50 kDa, ∼35 kDa, and ∼25 kDa), which were present at lower relative abundances in the other cultivations. Mass spectrometry analysis of the peptides from these three bands revealed that they predominantly consisted of four glycoside hydrolases: GH 7 (cellobiohydrolase), GH 5 (endoglucanase), GH10 (xylanase), and GH61 (copper-dependent polysaccharide monooxygenase) (Table 3). **Visual** comparison to *T. terrestris* demonstrated that there were no comparable prominent bands observed for the three cultivation conditions, which was consistent with the lower activity, **therefore the*T. terrestris*bands were not excised for analysis.**

**Table 3:** **Mass spectrometry analysis of major protein bands isolated from*T. aurantiacus*AFEX-SG secretome.**

**Prominent band**	**Description**	**Predicted MW**	**Peptides (95%)**^**1**^	**% Sequence coverage (95)**^**2**^
∼50 kDa	Possible GH 7 Cellobiohydrolase [GenBank: CAD79781]	48 kDa	250	72
∼35 kDa	GH 5 Endoglucanase [GenBank: CAZ67882]/GH10 Xylanase [GenBank: CAB65468]	37 kDa/36 kDa	249/176	72/65
∼25 kDa	GH 61 [PDB: 3ZUD_A]	24 kDa	60	93

^**1**^ Number of unique peptides matching protein with 95% confidence.

^**2**^ Sequence coverage of identified protein with unique proteins matching with 95% confidence.

## Discussion

In this work, we have shown that culture supernatant recovered from growth of *T. aurantiacus* on AFEX-SG exhibited high levels of saccharification activity on IL-SG. The higher levels of protein in the supernatants from the AFEX-SG grown supernatants may be due to additional nitrogenous compounds formed during the AFEX process [18]. The *T. aurantiacus* activity was 2–4 fold higher than the culture supernatant from *T. terrestris* at comparable protein amounts. CMCase activity was not predictive of saccharification activity, as supernatants from *T. aurantiacus* showed up to 11.5-fold lower activity on CMC compared to supernatants from *T. terrestris*. Cellobiohydrolase (pNPC) and β-glucosidase (pNPG) activities correlated with overall saccharification activities, demonstrating that exo-acting cellulases are critical for efficient saccharification of cellulose to glucose [19].

*T. aurantiacus* supernatant saccharification activities were comparable to the commercial enzymatic mixture Cellic CTec2 at 50°C **at similar protein mass loadings** and retained higher levels of activity up to 70°C. The temperature stability of the *T. aurantiacus* enzyme mixture is consistent with assays performed on purified endoglucanase and cellobiohydrolase enzymes from *T. aurantiacus* strains [4]. Cellic CTec2 has been shown to produce oxidized sugar products gluconic acid and cellobionic acid, suggesting the presence of GH61, a recently discovered Cu-containing cellulose monoxygenase expressed by many fungi, in the enzyme mixtures [20]. GH 61 from *T. aurantiacus* has been shown to reduce enzyme loadings 2-fold with Celluclast/N188 enzymatic cocktails from Novozymes and to be part of a cellulose oxidoreductase enzymatic system with cellobiose dehydrogenase that is synergistic with the well-studied hydrolytic system [13,15]. The presence of a prominent band in the corresponding to a GH61 protein at ∼25 kDA in SDS-PAGE analysis of the *T. aurantiacus* supernatants suggests that it may contribute to the high observed saccharification activity of *T aurantiacus* supernatants. The observed dominance of GH7, GH5, GH61 and GH10 in the AFEX-SG grown *T. aurantiacus* supernatants is similar to the GH profile of an optimal mixture of enzymes for glucose release from pretreated corn stover that was designed through computational modeling and high-throughput assays, a method referred to as GENPLANT [21]. The similarity between the GENPLANT-designed cocktail and the *T. aurantiacus* supernatants suggests that *T. aurantiacus* possesses very favorable properties for efficient cellulose hydrolysis.

Using chemical/UV mutagenesis and plate-based screeing, strains of *Trichoderma reesei* have been developed that secrete large amounts of cellulases and these strains are the basis for industrial production of cellulases for biomass deconstruction [22]. *T. aurantiacus* is an attractive candidate for a similar program of mutagenesis and screening, because it already secretes large amounts of cellulase enzymes, as demonstrated in the work described here, and it has a readily observed sexual state that could be used to select for specific mutations to enhance secretion of cellulases and remove deleterious mutations that accumulate during random mutagenesis and screening [23]. Therefore, *T. aurantiacus* has the potential to be a useful fungal species to understand cellulase secretion and biomass deconstruction under thermophilic conditions.

In conclusion, cultivation of *T. aurantiacus* and *T. terrestris* on biomass substrates revealed that *T. aurantiacus* grown on AFEX-pretreated switchgrass afforded high levels of cellulase enzymes in culture supernatants that were used to saccharify pretreated switchgrass at higher temperature and lower protein loadings than Cellic CTec2. Proteomic analysis of the supernatant demonstrated that the high activity could be attributed to the presence of high levels of proteins from the GH5, GH7, GH10 and GH61 families, which have been shown to be key enzymes in efficient cellulose hydrolysis. This work demonstrates that *T. aurantiacus* is a promising platform for the production of thermophilic enzymes for biomass deconstruction [1].

## Methods

### Materials

Fungal strains *Thermoascus aurantiacus* ATCC 26904 and *Thielavia terrestris* NRRL 8126 were obtained from American Type Cell Culture Collection. The fungal strains were maintained on potato dextorse agar (PDA) slants at 4°C. **Ethanol-washed** and [C_2_mim][OAc]-treated switchgrass (*Panicum virgatum* L.) were prepared as previously described [11]. Ammonia fiber expansion (AFEX)-treated and dilute acid-treated switchgrass were obtained as generous gifts and prepared using previously reported techniques [24,25]. Composition (cellulose:xylan:lignin content as percentage) of each biomass substrate is as follows: untreated switchgrass (35.1:20:21.2), IL-SG (51:21.4:18.5), AFEX-SG (36:20.8:20), and dilute acid-treated switchgrass (54:7.8:31.5). All chemicals, media, and other substrates were purchased from Sigma-Aldrich (St. Louis, MO) unless otherwise noted.

### Thermophilic fungal cultivation

For each fungal strain, spores from a PDA slant were used to inoculate 50 mL of potato dextrose broth (PDB) and cultured at 50°C, 150 rpm. The seed culture was used to inoculate 50 mL culture flasks with biomass substrate (10 g/L), corn steep liquor (**50% solids, concentrate of corn solubles**) (10 g/L), sodium chloride (2 g/L), potassium phosphate (5 g/L), calcium chloride dihydrate (0.1 g/L), magnesium chloride heptahydrate (0.5 g/L), zinc (II) sulfate heptahydrate (0.002 g/L), manganese (II) chloride tetrahydrate (0.008 g/L), iron (II) sulfate heptahydrate (0.001 g/L), copper (II) sulfate heptahydrate (0.006 g/L), cobalt (II) chloride hexahydrate (0.0002 g/L) and adjusted to pH 5 prior to autoclaving. Fungi were cultured at 50°C, 150 rpm for 3 days. After cultivation, cultures were filtered with Miracloth (EMD Millipore, Billerica, MA) to remove mycelia, followed by a 0.45-μm filter **with a cellulose acetate membrane (Thermo Scientific, Waltham, MA)** for spore removal. The clarified supernatant was stored at 4°C. Crude enzymes were produced in duplicate cultures and used for all subsequent experiments.

### Protein and glycoside hydrolase assays

Protein concentrations were determined using both Bradford assay (Bio-Rad, Hercules, CA) and bicinchoninic (BCA) assay (Pierce® BCA Protein Assay Kit, Thermo Scientific, Rockford, IL) methods using a 96-well plate (200 μL reaction volume) with bovine serum albumin as the standard. For BCA samples and standards, proteins were first precipitated with acetone and resuspended in 50 mM sodium acetate before measuring final concentration to remove interfering sugar residues [26]. All specific activities reported in this work were calculated using Bradford protein values. Endoglucanase and xylanase activities were assessed using the DNS (3,5-dinitrosalicylic acid) method using carboxymethylcellulose and birchwood xylan as substrates, respectively, with either glucose or xylose as the standard [27]. The enzyme reaction volume was 80 μL followed by 80 μL of DNS solution to measure released reducing sugars. One unit (U) of cellulase or xylanase activity was defined as the amount of crude protein releasing μmol of reducing sugar per min per mL of supernatant volume. Cellobiohydrolase (**pNPC**), β-d-glucosidase (**pNPG**), and β-d-xylosidase (**pNPX**) activities were determined using their respective *p*-nitrophenyl sugar substrates. 90 μL of sugar substrate was incubated with 10 μL of diluted enzyme, incubated for 30 min and quenched with 50 μL of 2% cold sodium bicarbonate. The absorbance of released *p*-nitrophenol was measured at 410 nm. Activities using *p*-nitrophenyl substrates were calculated as μmol *p*-nitrophenol released min^-1^ mg^-1^ crude protein. Due to possibility of color interference of the crude enzymes, samples were heat killed at 95°C overnight and used to remove background.

### Biomass saccharification

Saccharifications were performed in the presence of 2% (w/v) of AFEX-SG, IL-SG, or **dilute-acid pretreated switchgrass**. Each mixture was prepared in 50 mM sodium acetate, pH 5.0 with the designated amount of enzymes (g protein per g glucan in biomass), which were added after the reaction mixture was prewarmed to the reaction temperature to a final volume of 1 mL **in a 1.5 mL Eppendorf tube**. Saccharifications were carried out at specified temperatures in a shaker for 72 hours. In time-course studies on IL-SG (5 mL, performed in **15 mL Falcon tubes**), 500 μL samples were removed at designated time points. All hydrolysates were collected via centrifugation at 21,000x*g* for 5 min and 0.45-μm filtered to remove large biomass particles prior to sugar analysis. After filtration, samples were kept frozen at −20°C and thawed prior to analysis. Glucose and cellobiose concentrations were measured on an Agilent 1200 Series HPLC system equipped with an Aminex HPX-87H column (Bio-Rad) and Refractive Index Detector. Samples were run with an isocratic 4 mM sulfuric acid mobile phase. Sugar concentrations were determined using standards containing both glucose and cellobiose. Glucan conversion (mass of glucose plus cellobiose) was calculated using estimated glucan contents of pretreated switchgrass.

### Proteomic analysis of thermophilic fungal secretomes

For SDS-PAGE analysis, supernatants were concentrated 50-fold with 10,000 MWCO PES ultrafiltration column (Vivaspin 500, Sartorius, Germany), loaded onto an 8 – 16% Tris-glycine Mini gel (Invitrogen, Carlsbad, CA) and run at 130 V for 90 minutes. Gels were stained with GelCode Blue Safe Protein Stain (Thermo Scientific). After staining and extensive rinsing, target protein bands were excised and digested using trypsin [28].

Samples were analyzed on an ABSciex TripleTOF 5600 (ABSciex, Foster City, CA) coupled to Eskigent nano-lc systems (Applied Biosystems, Foster City, CA). Peptide samples were injected onto a Pepmap100 μ-guard column (Dionex, Sunnyvale, CA) via a Famos Autosampler (Dionex) and washed for 10 minutes with Buffer A (2% Acetonitrile, 0.1% Formic Acid) at 15 μL/min. Peptides were eluted onto an Acclaim Pepmap100 C18 column (75 μm x 150 mm, 300 nL/min flow rate; Dionex) via a gradient consisting of initial starting condition of 5% buffer B (98% Acetonitrile, 0.1% Formic Acid), increasing B to 10% in 2 minutes followed by a 58 minute ramp to 35% B. Subsequently, B was increased to 90% over 3 minutes and held for 7 minutes followed by a ramp back down to 5% B for 15 minutes to re-equilibrate the column to the original condition. Peptides were introduced to the mass spectrometer by using a Nanospray III source (ABSciex) operating in positive-ion mode (2400 V) and data acquired with Analyst TF 1.5.1. The TripleTOF 5600 was operated in information dependent acquisition (IDA) mode whereby the ten most intense ions within 400 m/z to 1250 m/z mass range exceeding 150 counts (charge states 2–5) were selected for MS/MS analysis (high sensitivity mode, UNIT resolution with rolling collision energy). MS/MS spectra were scanned from 100 m/z to 1600 m/z and were collected for a total accumulation time of 200 ms. Former parent ions were excluded for 16 seconds following MS/MS acquisition. The acquired spectra were exported as.mgf files from Peakview version 1.1.1 (ABSciex) and processed with Mascot version 2.3.02 with a peptide tolerance of ±50 ppm and MS/MS tolerance of ±0.1 Da; variable modification was Oxidation (M) with up to one missed cleavage for trypsin. As a limited number of protein sequences from *Thermoascus aurantiacus* are available, searches were performed against sequenced *T. aurantaicus* proteins available in GenBank combined with a full ORF database from *Thielavia terrestris* obtained through the Genome Portal of the Joint Genome Institute [29], a list of common contaminants (e.g., trypsin, human keratin) and consisted of 10,007 sequences. Mascot DAT files were uploaded into Scaffold version 3.5 and searched via X!Tandem with the same sequences and parameters.

## Abbreviations

AFEX: Ammonia Fiber Expansion pretreatment; BCA: Bicinchronic Acid; bG: beta-Glucosidase; [C2mim][OAc]: 1-ethyl, 3-methyl imidazolium acetate; CBH: Cellobiohydrolase; CMC: Carboxymethyl Cellulose; IL: Iionic Liquid pretreatment (in this work, 1-ethyl-3-methylimidazolium acetate); MCC: Microcrystalline Cellulose; SG: Switchgrass.

## Competing interests

The authors declare that they have no competing interests.

## Author’s contribution

SDM and SWS designed the experiments. SDM performed all experiments, analyzed data, and wrote the manuscript. TB and CP setup and performed proteomic sample analysis. SWS, BAS and PAD reviewed and edited the manuscript. All authors read and approved the final manuscript.
